# Progress in diagnosis and treatment of primary spondylodiscitis: a systematic literature review

**DOI:** 10.1530/EOR-2025-0041

**Published:** 2025-10-01

**Authors:** Dongdong Yu, Yongjian Kang, Wenxin Lu, Bin Chen

**Affiliations:** Department of Orthopedic Surgery, First Affiliated Hospital, Zhejiang University School of Medicine, Hangzhou, China

**Keywords:** primary spondylodiscitis, artificial intelligence, metagenomics next generation sequencing

## Abstract

**Objective:**

- Primary spondylodiscitis poses significant diagnostic and therapeutic challenges, with delayed diagnosis or improper treatment potentially resulting in severe complications. This systematic review aimed to summarize the latest diagnostic and therapeutic approaches for primary spondylodiscitis.

**Methods:**

- Adhering to PRISMA 2020 guidelines, we conducted a systematic literature review. PubMed was comprehensively searched for English-language original studies from January 1, 1990, to October 31, 2024. Structured queries combined keywords and MeSH terms relevant to spondylodiscitis, vertebral osteomyelitis, spinal infection, and associated treatments. Two reviewers independently screened titles, abstracts, and full texts, with manual bibliography searches as a supplement. A total of 147 articles were finally included.

**Results:**

- The literature indicates that diagnosis can be based on clinical suspicion, using serological, radiological, and microbiological tests. Newer methods such as metagenomics next-generation sequencing (mNGS) and positron emission tomography-computed tomography (PET-CT) can enhance diagnostic sensitivity and specificity. For confirmed cases, appropriate antibiotic therapy is crucial. Surgical treatment can benefit patients with neurological deficits, sepsis, spinal instability/deformity, epidural abscesses, or failed conservative treatment, accelerating recovery and reducing complications. Minimally invasive surgical approaches may also serve as an alternative to open surgery for select patients.

**Conclusion:**

- Although new technologies have improved diagnostic accuracy and treatment success rates for primary spondylodiscitis, establishing a robust staging system is vital to ensure patients receive effective, evidence-based treatment options.

## Introduction

Primary spondylodiscitis is infrequent in Western nations, with an incidence ranging from 1:100,000 to 1:250,000. Conversely, developing countries exhibit a higher incidence than their developed counterparts (1, 2). The annual rise in spondylodiscitis cases can be attributed to various factors, including an aging demographic, the prevalence of diabetes, Human Immunodeficiency Virus (HIV) infections, cancer therapies, and the extensive utilization of immunosuppressive medications (3, 4, 5, 6). Notably, diabetes and advanced age are significant risk factors. The infection rate is notably elevated between ages 60 and 80, with a threefold higher incidence in men compared to women (7, 8).

Diagnosing spondylodiscitis early is challenging due to its rarity and the atypical nature of clinical signs and symptoms, leading to a consistent delay in diagnosis. Delayed diagnosis results in severe consequences, including neurological deficits, vertebral instability, spinal deformity, or sepsis (9, 10, 11).

Likewise, spondylodiscitis presents controversies and treatment challenges. Diagnostic difficulties impede the selection of optimal anti-infective treatments. The inherent drug resistance in spondylodiscitis necessitates prolonged antibiotic courses, resulting in disease recurrence and drug toxicity accumulation. Unwise timing for surgery selection leads to delayed procedures and inferior surgical outcomes (12, 13).

Therefore, this article provides an overview of unresolved issues, aiming to identify protocols for accurate and timely diagnosis and treatment, thereby contributing to the effective management of spondylodiscitis. This article comprehensively addresses primary spondylodiscitis affecting the entire spinal column, encompassing the cervical, thoracic, lumbar, and sacral regions. While acknowledging anatomical and biomechanical variations across spinal segments, we focus on the shared diagnostic and therapeutic principles of this condition, while highlighting segment-specific considerations, particularly regarding surgical approaches and neurological risks where applicable.

## Methods

We conducted a systematic literature review in accordance with the PRISMA 2020 guidelines (14). Our comprehensive searches spanned PubMed from January 1, 1990, to October 31, 2024. We utilized structured queries that incorporated keywords and MeSH terms. These terms were related to spondylodiscitis, vertebral osteomyelitis, spinal infection, and their associated treatments, such as ‘conservative treatment’ or ‘surgical intervention’.

Following are the inclusion criteria for the selected studies: i) only empirical studies, peer-reviewed studies, and journal publications were included. ii) Studies were included from January 1, 1990, to October 31, 2024. iii) Studies were included only if they were in the English language. iv) Studies were included only if they related to primary spondylodiscitis as a specific cohort.

Studies were excluded if they met the following criteria: i) conference proceeding papers, general magazine articles, commentaries, opinion pieces, newsletters, and any gray or secondary literature were excluded. ii) Studies were excluded if they were not in the English language. iii) Studies that were not about primary spondylodiscitis were excluded.

**Figure 1:**
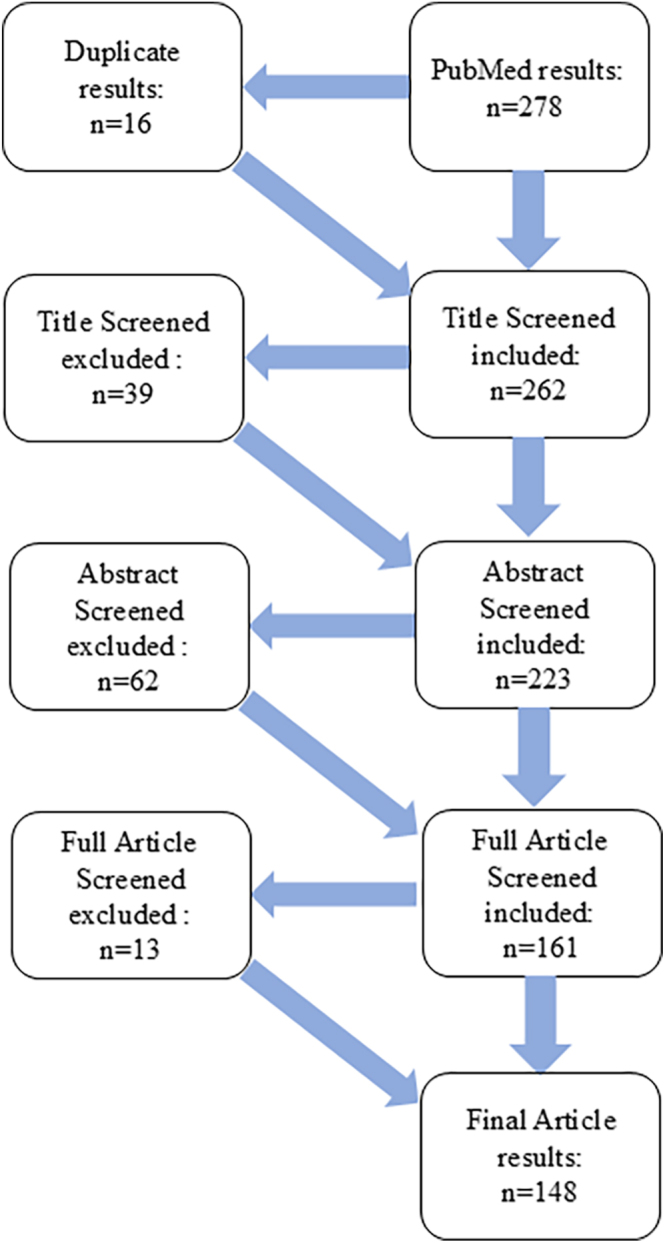
PRISMA flow chart of the systematic literature review for primary spondylodiscitis.

Two reviewers worked independently to screen the titles and abstracts, as well as the full texts of the articles. To ensure a thorough search, we also manually searched bibliographies for additional relevant studies. We maintained a record of the selection process, including the reasons for exclusions. This information was then summarized in a PRISMA flow diagram, which is presented as Fig. 1.

## Results

This review encompassed a comprehensive analysis of 148 included studies. The distribution of research methodologies was diverse: quantitative designs predominated (92 studies, 62.2%), including retrospective cohort studies, randomized controlled trials (RCTs), diagnostic tests, and meta-analyses. Qualitative methodologies were employed in 43 studies (29.1%), comprising systematic reviews, clinical guidelines, and case reports. A further 13 studies (8.8%) utilized mixed-methods approaches.

Geographically, the studies demonstrated a wide international scope with shifts in regional contributions: research originating from China constituted the largest single-country cohort (43 studies, 29.1%), followed by the United States (38 studies, 25.7%) and various European countries collectively (38 studies, 25.7%).

The included studies were predominantly published in high-impact journals. The vast majority (100 studies, 67.6%) appeared in JCR Q1 journals. A significant portion was published in Q2 journals (33 studies, 22.3%), while Q3/Q4 journals accounted for 15 studies (10.1%).

Publication dates revealed a strengthened recent focus: 74 studies (50.0%) were published between 2020 and 2024, peaking in 2023 with 33 studies. The research focus evolved over time, shifting from foundational pathology and open surgery towards diagnostic standards, optimized conservative management, and, increasingly, minimally invasive techniques and AI applications in the most recent period.

Thematic analysis showed a primary emphasis on treatment strategies (75 studies, 50.7%), covering surgical approaches and antibiotic regimens. Diagnostic technologies were the focus of 49 studies (33.1%), including advancements in imaging, biopsy techniques, and metagenomic next-generation sequencing (mNGS). Emerging technologies, particularly AI and endoscopic applications, were explored in 24 studies (16.2%).

## Discussion

The in-depth analysis of the reviewed articles was conducted under different thematic categories of primary spondylodiscitis. The results of this thematic analysis will be presented separately in the following text, followed by an overall discussion.

### Etiology

Primary spinal infections can be broadly categorized as pyogenic or non-pyogenic. Pyogenic infections are typically caused by bacteria such as Staphylococcus aureus and Escherichia coli, which induce acute suppurative inflammation. In contrast, non-pyogenic infections encompass granulomatous pathogens, including Mycobacterium tuberculosis (MTB), Brucella spp., and fungi, which often present with an indolent course. This classification guides the subsequent pathogen-specific review.

#### Tuberculous spondylodiscitis

MTB, a representative non-pyogenic pathogen, is one of the leading global causes of spondylodiscitis. A systematic review of 1,060 cases with microbiologic details found that 30.9% were attributed to MTB (15). Its prevalence varies regionally, with a global prevalence of 46% in developing countries (16). Notably, in a published case series evaluated by positron emission tomography-computed tomography (PET-CT) scanning, spinal tuberculosis showed noncontiguous spinal involvement in 63.6% of cases (17).

#### Non-tuberculous spondylodiscitis

Staphylococcus aureus is the predominant non-tuberculous pathogen. The systematic review indicated that 40.3% of cases were attributed to Staphylococcus spp., and 28.3% to other bacterial pathogens; fungal (0.5%) and viral (0.1%) etiologies were relatively rare (15). The prevalence of pathogens varies regionally due to local disease infection patterns. In areas where brucellosis is endemic, such as the Middle East and the Mediterranean basin, it is the leading cause of spondylodiscitis, with reported prevalence rates ranging from 21 to 48% (18).

Regardless of pyogenic or non-pyogenic classification, the thoracolumbar and lumbar regions of the spine are the most commonly affected, while the lumbosacral and upper cervical thoracic segments are less frequently involved (12, 19, 20). This increased vulnerability can be attributed to the dynamic stability of the spinal column, especially at the thoracolumbar junction, which is prone to microtrauma. Such microtrauma creates an environment conducive to bacterial proliferation and dissemination. Pathogenic bacteria predominantly reach the spinal bones via hematogenous spread (21). Frequently, two adjacent vertebrae are simultaneously affected due to their shared blood supply from the same segmental artery. Moreover, bacteria may spread through Batson’s plexus, leading to focal vertebral lesions and implicating noncontiguous vertebrae. Conversely, dissemination via the anterior or posterior longitudinal ligaments can result in the involvement of a large number of contiguous vertebrae (22).

Protracted delays in diagnosis and treatment are associated with a poor prognosis (23, 24, 25). Injuries to spinal mobile segments can lead to instability, reduced mobility, and neuromedullary compression, often necessitating surgical stabilization (26, 27). The Infectious Diseases Society of America (IDSA) clinical practice guidelines highlight five interrelated factors crucial for refining decision-making in patients with spondylodiscitis: the presence of infectious agents, the degree of segmental instability, abscess formation, the extent of neurologic impairment, and the presence of infected lesions (28). The severity of infection in spondylodiscitis patients is closely correlated with the pathogenicity and virulence of the bacteria, the systemic nature of the disease, and the site of infection origin (cardiac, gastrointestinal, or dental-oral) (29).

Therefore, timely and systematic assessment is essential for the accurate diagnosis and effective management of this disease.

### Diagnosis

The diagnostic approaches discussed below are applicable to spondylodiscitis regardless of spinal level, although imaging interpretation and biopsy access may vary technically.

Due to the widespread occurrence of lumbar back and radicular pain, diagnosis is frequently delayed, as indicated by a mean diagnostic duration of 2–4 months in a study (30). In another study, initial misdiagnosis occurred in 34% of 101 patients with spinal infections (31). Even paraplegic patients may be delayed in diagnosis (32). Patients with spondylodiscitis exhibit a notably lower incidence of febrile symptoms compared to other infectious diseases, with only 45% of those with bacterial spinal infections experiencing fever. MTB, Brucella abortus, or fungal infections seldom manifest with fever (33, 34, 35).

Laboratory diagnosis of patients with spondylodiscitis includes white blood cell count, erythrocyte sedimentation rate, and C-reactive protein (CRP). While they serve as diagnostic references, they lack sufficient sensitivity and specificity. Elevated CRP values are present in more than 90% of spondylodiscitis cases, so significant elevation of CRP is the most reliable marker (36). Furthermore, the CRP-to-albumin ratio is a valuable metric for monitoring spinal epidural abscesses (37).

Imaging evaluation of patients with spondylodiscitis includes X-rays, computed tomography (CT) scans, and magnetic resonance imaging (MRI) (38, 39). MRI is considered the diagnostic gold standard for spondylodiscitis, offering a clear visualization of the infection’s extent and severity. Characteristic MRI findings in spondylodiscitis include: i) hypointensity of the vertebral bodies and intervertebral discs with poorly defined endplate margins on T1-weighted imaging (T1WI); ii) hypointensity of the vertebral bodies and discs with indistinct endplate boundaries on T2-weighted imaging (T2WI) or Short Tau Inversion Recovery (STIR) sequences; and iii) increased contrast enhancement of the vertebral bodies and discs. However, it is essential to differentiate these MRI features from those related to Modic changes, acute Schmorl nodes, disc herniation, malignancy, or acute endplate injuries (40). CT is crucial in diagnosing specific patients unsuitable for MRI, including patients with pacemakers, metal implants, or claustrophobic compartment syndrome. Moreover, percutaneous puncture biopsy by three-dimensional CT reconstruction images and CT-guided is an essential way to obtain tissue from lesions (41, 42). While emission computed tomography (ECT) and PET-CT match MRI’s sensitivity and provide greater specificity (11, 43, 44, 45, 46, 47), due to their cost-effectiveness and accessibility, the current recommendation from IDSA guidelines is to utilize PET-CT only when MRI is contraindicated (28).

Isolating pathogenic microorganisms is essential for spondylodiscitis management, guiding the selection of appropriate antibiotics (48). Blood cultures identify pathogens in approximately 40–60% of cases. In cases with negative blood cultures and no severe infection or neurologic signs, a radiologically guided percutaneous biopsy of the affected area is advised (8, 49). For patients with negative cultures and initial biopsy results, a repeat biopsy is effective in 60% of instances (50). Comparatively, the diagnostic rates for blood cultures, percutaneous puncture biopsies, and surgical specimen cultures were 43.6, 72.7, and 91.6%, respectively (51). Next-generation sequencing (NGS) is helpful in patients who have received prior antibiotic therapy or who are infected with pathogens that cannot be easily cultured, such as Brucella or Mycobacterium (52). In focal complications of brucellosis, the application of blood cultures and two serologic diagnoses can be performed in >95% of cases (53).

Recent advancements in NGS have significantly addressed the microbiologic diagnostic challenges of spondylodiscitis (54). Research has showcased the effectiveness of blood macro-genomic NGS (referred to as ‘liquid biopsy’) in identifying pathogens that traditional methods might overlook, thus obviating the necessity for expensive and invasive surgical sampling procedures (52, 55, 56). NGS is a high-throughput, scalable, and rapid sequencing technology. It identifies microorganisms by sequencing the entire genomic content of a clinical sample, thus bypassing the need for traditional pathogen culture. This capability allows for the detection of fastidious bacteria that are difficult to cultivate, including non-tuberculous mycobacteria (57, 58). mNGS, grounded in advanced DNA sequencing technology, grants access to millions of DNA strands, facilitating the identification of over 1,400 species. This process has significantly reduced detection time to 1–2 days (59). Moreover, the establishment of a comprehensive single nucleotide polymorphism database is feasible, enabling the identification of any strain. This unbiased and hypothesis-free approach is particularly well-suited for establishing microbiological diagnoses in scenarios where conventional cultures and serological methods are insufficient for detecting microorganisms (60, 61). Nevertheless, the current absence of specific pathogen libraries for spondylodiscitis, unlike other well-established infectious sites, hampers the precise and swift detection capability of NGS in spondylodiscitis. In a recent multicenter study involving ten children with spinal discitis, all ten patients had negative blood cultures. However, applying plasma mNGS to detect Kingella resulted in limited antimicrobial coverage in nine patients, eight of whom were diagnosed without the need for biopsy. While mNGS shows promise in revolutionizing infectious disease diagnosis, its theoretical potential has not been fully realized as of yet (62).

Currently, AI contributes to the diagnosis and risk prediction of spinal infections. AI technology can analyze spinal imaging through deep learning algorithms to rapidly identify infected areas and differentiate between types of spinal infections (63, 64, 65, 66, 67). This approach enhances diagnostic accuracy. In addition, AI models can predict spinal infection risks by analyzing patients' clinical data (68, 69). Detailed information is provided in Table 1.

**Table 1:** A summary of research on the application of artificial intelligence to spinal infections.

Study	Study population	Research purpose	Research variables	Methodologies	Results, AUC
Feng *et al.* (68)	TS patients	Predicting SCI	Clinical characteristics	RF model	0.816
Chen *et al.* (67)	TS or BS patients	Differentiate TS from BS	MRI	VGG19	0.973
Oz Haim *et al.* (64)	Patients with various spinal pathologies	Differentiate between spinal pathologies	Preop MRI and post-op pathological results	A deep learning model built on the Fast.ai framework on top of the PyTorch environment	0.78
Zhang *et al.* (69)	Spine surgery patients	Predicting postoperative SSI following spine surgery	Clinical characteristics	NBM	0.95
Shuo *et al.* (65)	TS & SM patients	Discriminating between spinal TS and SM	MRI	MViTv2	0.98
Mukaihata *et al.* (66)	PS & SMC patients	Differentiate spinal pyogenic spondylitis from modic change	MRI	CNN	0.95
Liu *et al.* (63)	Postop SSI after LSS patients	Predict SSI risk in patients after LSS	Clinical characteristics	GBM	0.923

TS, tuberculous spondylitis; BS, brucellar spondylitis; SSI, surgical site infection; LSS, lumbar spinal surgery; SM, spinal metastases; SCI, spinal cord injury; PS, pyogenic spondylitis; SMC, spinal modic change; RF, random forest; NBM, Naive Bayes Model; MViTv2, Multiscale Vision Transformers; MRI, magnetic resonance imaging; CNN, convolutional neural network; GBM, gradient boosting machine; VGG19, Visual Geometry Group 19-layer Network.

### Therapy

The management of spondylodiscitis remains a contentious issue, encompassing the optimal timing for antibiotic therapy and the criteria and methods for surgical intervention (70). The treatment modalities for spondylodiscitis are broadly classified into conservative and surgical approaches, with a focus on identifying the causative microorganisms. The foundational principles of treatment are aimed at eradicating the infection, restoring and maintaining spinal structure and function, and mitigating symptoms (28).

#### Antibiotic therapy

If the patient stays stable, antibiotics should be initiated following the collection of a culture sample. In critically ill patients or those with negative culture results, intravenous empiric broad-spectrum antibiotic therapy is indicated, commonly consisting of third-generation cephalosporins or fluoroquinolones in conjunction with clindamycin or vancomycin (71). Empiric antimicrobial therapy must encompass Gram-positive bacteria, including Methicillin-resistant Staphylococcus aureus and streptococci, and Gram-negative bacilli, primarily E. coli (72). Consideration of the drug’s tissue concentration is crucial in antibiotic selection, favoring those with substantial concentrations in bone and soft tissues (73).

The duration of antibiotic therapy remains controversial and is influenced by the patient’s immune status, pathogenic bacterial species, resistance, and internal fixation status (74). Septic infections demand continuous antibiotic therapy, typically involving a minimum of 6 weeks of parenteral or oral antimicrobials. A pivotal randomized controlled trial demonstrated that 6 weeks of therapy is non-inferior to 12 weeks for achieving clinical cure at 1 year in patients with microbiologically confirmed pyogenic vertebral osteomyelitis, suggesting the standard duration can be reduced for many patients (75).

Surgical clearance can shorten parenteral antibiotic use in select patients (76, 77). Septic spondylodiscitis with severe complications may necessitate an extended duration (78). Tuberculous spondylodiscitis is treated with first-line drugs involving isoniazid (H), rifampicin (R), pyrazinamide (Z), ethambutol (E), and streptomycin. Initial administration of four drugs (HRZE) for 2 months rapidly eliminates most organisms. This is followed by a ‘continuation period’ involving two drugs (HR) for 4 months to consolidate treatment. However, the 2010 World Health Organization revision recommends a 9-month duration for antituberculosis treatment due to the high risks associated with disability and mortality and challenges in assessing treatment response (79, 80, 81). Traditionally, the preoperative period involves four cycles of chemotherapy over 2–4 weeks. However, recent clinical practice supports short-term, intensive preoperative chemotherapy regimens over 1–7 days as feasible (82). A recent study demonstrates comparable outcomes with 6 months of antituberculosis treatment to the conventional 12-month regimen in up to 24 months of follow-up (83).

Brucella infections are primarily treated with doxycycline and rifampicin, potentially with aminoglycosides. Complicated cases may require more prolonged antibiotic courses compared to uncomplicated cases (84). Spinal fungal infections necessitate suitable antifungal therapy, with the specific antifungal drug choice contingent on the fungus. The optimal duration of antifungal therapy lacks consensus. The IDSA guidelines for Aspergillus infections recommend a minimum treatment duration of 8 weeks, although treatment periods exceeding 6 months are often required (85).

#### Surgical management

The surgical management of spondylodiscitis remains controversial, attributable to the absence of a standardized classification system and universally acknowledged surgical guidelines (86). Despite the non-necessity of surgical treatment in the majority of spondylodiscitis cases, approximately 50% of affected patients ultimately undergo surgical treatment (87). Surgical treatment objectives encompass the removal of the infected lesion, identification of the infecting microorganism, stabilization of the affected spinal segment, and facilitation of osseointegration. Furthermore, surgical intervention enables early postoperative mobilization and offers a more definitive and reliable approach to treating spinal deformities. Timely surgical treatment is advisable for patients exhibiting progressive neurological deficits, deformities, and spinal instability, irrespective of the presence or absence of pain, and with or without appropriate antimicrobial drug therapy. Surgical intervention is advised for patients with persistent or recurrent bloodstream infections without an identifiable alternative source or those experiencing escalating pain despite receiving appropriate pharmacotherapy, with or without the presence of spinal instability (24, 28, 88, 89).

Spinal stability serves as a crucial determinant in guiding surgical decisions for spondylodiscitis. Similar to metastatic spinal tumors, spondylodiscitis results in the chronic instability of the spine, distinguishing it from the acute instability induced by traumatic injuries (90). Recent investigations suggest the effectiveness of extrapolating the spinal instability neoplastic score (SINS) to evaluate instability in spinal tuberculosis. However, certain parameters of the SINS prove inapplicable to spondylodiscitis (91, 92). A study employed the spinal instability severity score with high confidence and validity to identify unstable lesions in spondylodiscitis. This approach holds the potential for evaluating treatment options grounded in spinal biomechanics (93).

Optimal treatment selection for spondylodiscitis involves considerations beyond spinal stability; aspects such as overall patient health, neurological status, and patient preferences necessitate thorough evaluation (94, 95). Epidural abscesses are an important cause of neurologic deficits (96). Surgery is strongly advocated for cervical and thoracic epidural abscesses, regardless of nerve damage, due to the swift progression of neurological impairment (97, 98, 99). Conversely, the decision to undertake surgery for lumbar epidural abscesses is contingent upon various factors, including the patient’s neurologic symptoms, underlying health conditions, the severity of the infection, and their overall clinical status. Urgent surgical intervention is warranted in instances of spinal cord compression and the progression of neurologic deficits (100, 101, 102). Timely surgical intervention enhances the prognosis in terms of the quality of survival (103, 104). Delaying emergency surgery beyond 24–36 h post-onset of neurologic deficits may result in permanent impairment of neurologic function (105).

The general health of the patient has a significant impact on the decision to operate for spondylodiscitis. For critically ill patients with spondylodiscitis, delayed surgery (performed 3 days after admission), especially between 10 and 14 days post-admission, is associated with lower mortality. These findings highlight the potential benefits of considering surgical timing to improve patient outcomes (106). In elderly patients, for example, patients are usually immunocompromised, have impaired physical functioning, and have multiple co-morbidities, and are therefore usually treated conservatively (107). However, this approach requires prolonged bed rest, which may lead to complications such as deep vein thrombosis and pulmonary infections that can seriously affect quality of life (108). With further progression, abscess formation may lead to vertebral destruction or nerve injury (109). Therefore, the goal of surgical intervention in elderly patients with infectious spondylodiscitis is to enable early mobilization and reduction of complications. Research indicates that for thoracolumbar spondylodiscitis, one-stage posterior debridement, decompression, interbody fusion, and internal fixation are deemed safe and effective treatments for elderly individuals with spondylodiscitis (110). Conversely, in elderly individuals with cervical spondylodiscitis, both anterior cervical diskectomy and fusion and laminectomy are viable and safe treatment strategies (111).

Immunocompromised patients, including those with Acquired Immune Deficiency Syndrome, organ transplantation, a history of intravenous drug abuse, prolonged steroid therapy, hematologic malignancies (112), chronic liver disease (113), or solid organ transplants (114), experiencing spondylodiscitis face delayed diagnosis owing to a diminished immune response in the host and the absence of early, typical signs and symptoms, which may include unreliable inflammatory marker elevation in transplant recipients. This situation poses challenges for physicians in disease management (115). These patients might harbor diverse pathogens (Gram-negative bacteria, pseudomonas, fungal infections such as Aspergillus species, and Salmonella), compared to the healthy population, with pathogen profiles varying significantly based on the specific immunocompromising condition. However, HIV treatment with highly active antiretroviral therapy reduces infection rates and does not alter the bacteriologic distribution, unless the patient exhibits low CD4+T cell levels (116). Critically, regardless of the underlying cause of immunosuppression, these patients demonstrate higher rates of neurological compromise at presentation and are more likely to require surgical intervention, and the likelihood of severe osteomyelitis, with or without an epidural abscess, is increased (5, 62, 116, 117). Timely surgical treatment can identify the causative pathogen, stabilize the affected spinal segment, ensure proper abscess drainage, and foster fusion (118). Postoperative complication rates are notably elevated in this population. Given the immune deficiency, postoperative antibiotic administration should be standardized, meticulously regimented, and often prolonged (5). Furthermore, these patients face significantly higher short- and long-term mortality risks compared to immunocompetent individuals.

Surgical access and method should be tailored to the pathogen’s characteristics, lesion location, and nature (119, 120). For fluid-occupying lesions within the intervertebral space or spinal canal, the widely accepted approach involves laminotomy and minimally invasive drainage techniques, complemented by thorough irrigation. Employing this strategy effectively minimizes trauma and facilitates comprehensive lesion removal, thereby reducing the risk of potential complications. Conversely, the presence of solid space-occupying lesions, such as necrotic tissue and granulation tissue, may require more extensive surgical intervention. Recommendations often include spinal canal decompression and/or intervertebral laminectomy to maximize the removal of diseased tissue, facilitating subsequent reconstruction and internal fixation (102, 121).

In thoracolumbar spondylodiscitis, one-stage posterior decompression and debridement with internal fixation typically yield favorable clinical outcomes, encompassing effective infection control, relief from back pain, correction of kyphosis, and partial or solid fusion (122, 123, 124). When achieving adequate clearance proves challenging through posterior surgery alone, particularly in cases involving vertebral ventral abscesses, a combination of anterior and posterior surgery may be employed. However, anterior spinal surgery carries a significant risk of complications and mortality (37, 125). In instances where two-stage surgery is necessary, posterior internal fixation and fusion surgery can be employed as the second stage following thorough drainage and debridement in the initial stage. Common bone implantation methods for spondylodiscitis include autogenous granulated bone graft, autogenous block bone graft, and titanium mesh bone graft (126). In cases where significant spinal deformity is present and autologous bone reconstruction alone lacks stability, the employment of a titanium mesh bone graft is recommended, proving effective in restoring the spinal Cobb angle (127). Cervical spondylodiscitis combined with spinal epidural abscess is considered a serious spinal condition. One-stage radical debridement and reconstruction have gained widespread acceptance, with surgical approaches categorized as simple anterior, combined anterior, and simple posterior. Notably, favorable clinical outcomes are attainable for most cervical spondylodiscitis cases through simple anterior debridement and internal fixation (99, 128). In addition, endoscopic-assisted anterior surgery effectively mitigates the risks associated with anterior surgery, demonstrating efficacy in achieving thorough debridement and spinal fusion (99, 129).

Minimally Invasive Spine Surgery (MISS) has profoundly influenced spondylodiscitis treatment, offering patients cost-effective and advantageous surgical treatment. The safety and efficacy of thoracoscopic access in the management of spondylodiscitis in the thoracic spine segment have been demonstrated at certain institutions (130, 131). For patients with single-segment thoracolumbar suppurative spondylodiscitis or those with high functional expectations, percutaneous screw-rod internal fixation is suggested as an alternative to bracing or as a standard procedure following anterior debridement (132, 133, 134). Studies have demonstrated the efficacy of minimally invasive posterolateral transforaminal approaches in achieving thorough clearance of intervertebral space infections. These approaches, when combined with percutaneous pedicle screw fixation, contribute to additional spine stabilization through MISS (124). Furthermore, percutaneous endoscopic drainage has been documented as an efficacious alternative to open surgery for managing internal abscesses (135). Detailed information is presented in Table 2. Especially, a recent retrospective multicenter study utilized a database of 70 patients from five European neurosurgical centers (136). Patients with primary cervical spondylodiscitis underwent a minimally invasive surgical approach via limited funnel-shaped cervical microdiscectomy. This involved a 4-mm incision of the anterior longitudinal ligament and a 6-mm incision of the posterior longitudinal ligament, followed by PUS drain placement postoperatively, extensive irrigation of the intervertebral and epidural spaces, but no fusion. Among the 70 patients, inflammatory markers normalized within 8–12 weeks, with only one recurrence. After a mean follow-up of 48 months, all patients achieved complete recovery without neurological deficits, spinal instability, or kyphosis. Radiological examinations confirmed bony fusion with no infection recurrence. MISA provides a valuable, stable, and less invasive option for treating CSD, enabling effective microbial identification and spinal cord decompression, leading to favorable patient outcomes.

**Table 2:** Application of minimally invasive spinal surgery in the treatment of spinal infections.

Disease type	Cases, *n*	Surgical procedure	Result	Study
Spinal TB	16	VATS	88% had good neurologic recovery	Kapoor *et al.* (130)
Spinal TB	23	VATS	94% had neurologic recovery	Jayaswal *et al.* (142)
Spinal TB	15	VATS	Average neurological recovery was 1.44 grades on the scale by Frankel *et al.*	Zheng *et al.* (149)
Thoracic or lumbar TB	42	PPS fixation	Pain decreased; less operative time, blood loss and shorter hospital stay	Guo *et al.* (132)
PS	125	PPS fixation	Less operative time, blood loss and shorter hospital stay	Janssen *et al.* (134)
Single segment lumbar TB	60	OLIF	Less operative time, blood loss and shorter hospital stay	Du *et al.* (143)
Lumbar TB	22	PPS fixation and minimally invasive LSILD and bone graft fusion	Less bleeding, less trauma, and faster recovery	Chen *et al.* (144)
IS	17	PEDD	82% had good neurologic recovery	Choi *et al.* (135)
IS	24	Percutaneous endoscopic surgery	No spinal instability	Lin *et al.* (145)
IS	60	Endoscopic surgery	2 (3.3%) experienced infection relapse	Lin *et al.* (146)
High-risk patients with spondylodiscitis	14	FEDD	Significant improvement in symptoms	Lin *et al.* (147)
IS	28	PTD	Immediate pain relief rate: 75%; long-term FU success rate: 68%	Hadjipavlou *et al.* (148)

PS, pyogenic spondylodiscitis; LSILD, lateral small incisions lesion debridement; IS, infectious spondylodiscitis; TB, tuberculosis; FU, follow-up; VATS, video-assisted thoracoscopic surgery; pps, percutaneous pedicle screw; OLIF, oblique lateral interbody fusion combined with percutaneous pedicle screw fixation; PEDD, percutaneous endoscopic debridement and drainage; FEDD, full endoscopic debridement and drainage; PTD, percutaneous transpedicular discectomy.

### Improvement in clinical algorithm of primary spondylodiscitis

**Figure 2:**
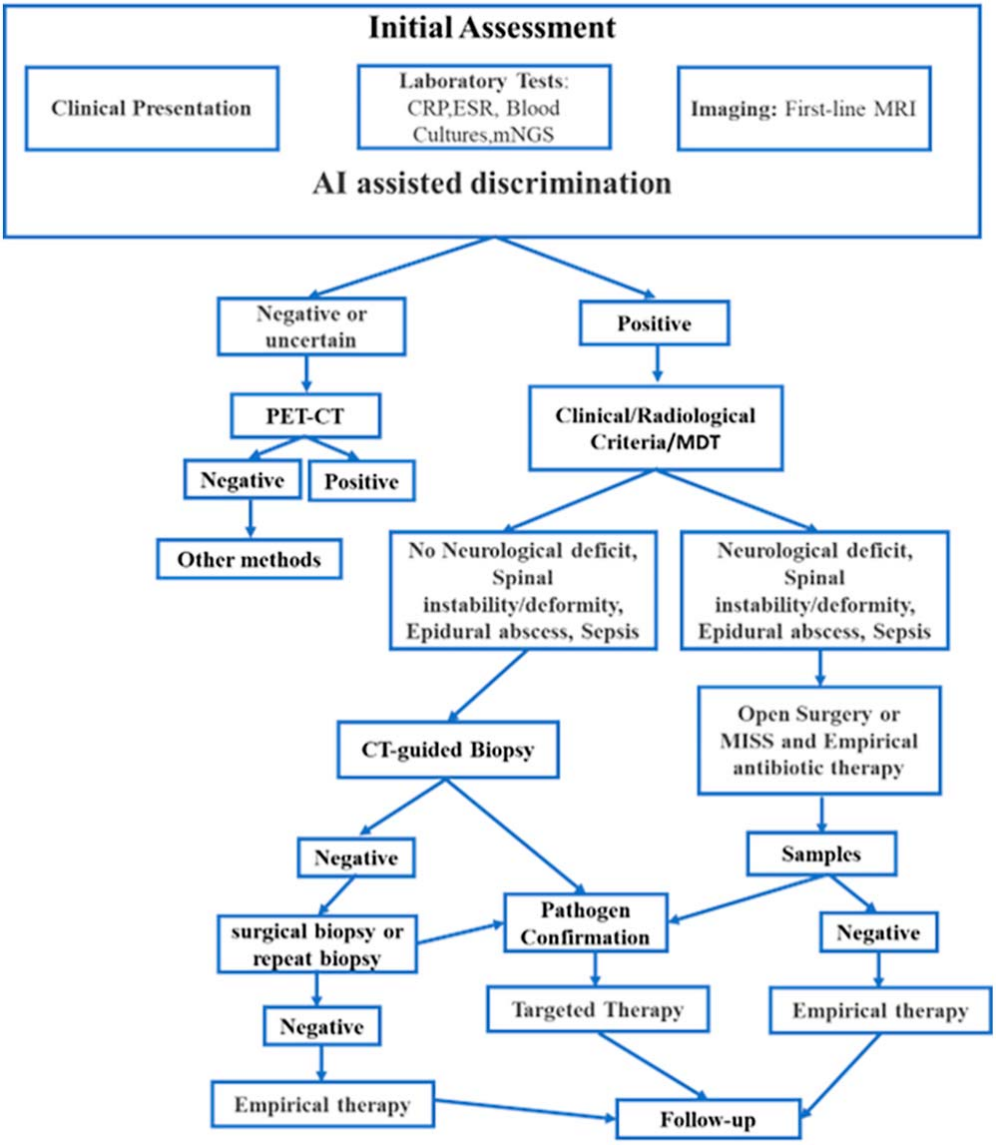
A proposed algorithm for the treatment of primary spondylodiscitis (CRP, C-reactive protein; ESR, erythrocyte sedimentation rate; WBC, white blood cell; MRI, magnetic resonance imaging; CT, computed tomography; PET-CT, positron emission tomography-computed tomography; MISS, minimally invasive spine surgery; mNGS, metagenomic next-generation sequencing; MDT, multidisciplinary treatment).

The primary spondylodiscitis diagnosis and treatment process proposed in this review (Fig. 2) is an optimized process based on international guidelines (such as the 2015 IDSA guidelines) (28), combined with current research progress and focusing on the pain points in current diagnosis and treatment. The improvements are mainly reflected in the following aspects.

First, unlike the current guidelines that use mNGS as a salvage method after negative culture results, this algorithm incorporates mNGS in parallel with routine culture for initial pathogen detection, and simultaneously introduces AI-driven imaging analysis.

Second, unlike the IDSA guidelines that recommend PET-CT only when MRI is contraindicated, this process emphasizes the importance of PET-CT when the initial assessment is uncertain, thus avoiding repeated invasive procedures.

Third, a multidisciplinary team (MDT) decision-making node has been established, requiring all confirmed patients to be jointly assessed by infectious disease, spinal surgery, imaging, and microbiology physicians before treatment initiation (137, 138).

Finally, MISS has been upgraded from a supplementary to a first-line option, with clear indications (single-segment lesions, high functional demands, and multiple comorbidities).

The core innovation of this algorithm lies in creating a closed-loop system of ‘precise diagnosis, intelligent decision, minimally invasive intervention’. Early molecular diagnosis (mNGS) and AI imaging solve the problem of delayed diagnosis. The MDT mechanism enables rapid and effective treatment decisions. MISS technology integration promotes a shift in surgical intervention from ‘trauma control’ to ‘function preservation’. This provides an operable clinical framework for establishing a spinal infection staging system and is expected to be a key reference for future guideline updates.

## Summary and outlook

Spondylodiscitis is an infrequent and impactful condition associated with severe consequences, including spinal deformity, nerve damage, and paralysis, often exacerbated by delayed diagnosis and treatment. The adoption of standardized and timely conservative or surgical interventions proves instrumental in mitigating these risks. The novel technologies further enhance the diagnostic and therapeutic landscape for spondylodiscitis. mNGS serves as a swift and effective tool, utilizing high-throughput data for the precise identification of pathogenic bacteria, thereby guiding antibiotic treatments. PET-CT, serving as a valuable adjunct to MRI, exhibits commendable accuracy in early-stage diagnosis, demonstrating superior sensitivity and specificity. The pervasive application of AI, particularly through machine learning, holds significant promise across the realms of diagnosis, prevention, and prognostic assessment of spondylodiscitis (63, 139, 140, 141). MISS enables debridement, drainage, internal fixation, and fusion with reduced trauma. Despite the innovative technologies, additional clinical research is imperative to establish a robust staging system for spondylodiscitis, ensuring the provision of effective, evidence-based medical treatment options for patients.

## ICMJE Statement of Interest

The authors declare that there is no conflict of interest that could be perceived as prejudicing the impartiality of the work reported.

## Funding Statement

This research was supported by Zhejiang Provincial Natural Science Foundation of China under Grant No. LQ22H100003 The Medical Health Science and Technology Project of Zhejiang Provincial Health Commission No. 2020KY132 and The Zhejiang Province Leading Geese Plan No. 2025C02170.
